# Interaction between genetic factors, *Porphyromonas gingivalis* and microglia to promote Alzheimer’s disease

**DOI:** 10.1080/20002297.2020.1820834

**Published:** 2020-09-16

**Authors:** Ingar Olsen, Sim K. Singhrao

**Affiliations:** a Institute of Oral Biology, Faculty of Dentistry, University of Oslo, Oslo, Norway; b Brain and Behavior Centre, Faculty of Clinical and Biomedical Sciences, School of Dentistry, University of Central Lancashire, Preston, UK

**Keywords:** Microglia, immune cells, inflammation, brain, training, tolerance, hyperactivity, *P. gingivalis*

## Abstract

In late-onset Alzheimer disease (AD) pathogenesis, genes, infections and immunity could be significant factors. We have reviewed if the keystone periodontal pathogen *Porphyromonas gingivalis* may affect genes and microglia (primary immune cells in the brain) to promote AD development. Genes for apolipoprotein, clusterin, CD33, triggering receptor expressed on myeloid cells-2 (TREM-2), tyrosine kinase binding protein (TYR-OBP), and complement receptors can affect microglia. Most of these genes can also be affected by *P. gingivalis* via its mastering of immune suppression. Besides, *P. gingivalis* can affect microglia directly in several ways. Taken together, genetic predisposition, *P. gingivalis* infection and microglia could promote neurodegeneration typical of that reported for AD.

## Introduction

Amyloid-beta (Aβ) plaque and phosphorylated tau (p-Tau) binding to neurofibrillary tangles (NFTs) are important neuropathological and diagnostic markers of Alzheimer’s disease (AD). Both lesions in their diverse peptide sizes (Aβ and p-Tau) can act as toxins in and outside cells *in vitro* and *in vivo* animal models [1–4]. A second factor of AD is brain infection/inflammation where the keystone pathogen in ‘chronic’ periodontitis *Porphyromonas gingivalis*, seems to play an important role. Cerebral inflammation in the form of activated microglia is a third major histopathological feature, but without a role in the neuropathological diagnosis of AD.

## Aim

The aim of the present review is to discuss how genetic factors, *P. gingivalis* periodontal infection and microglia can interact to promote AD. Potential mechanisms for microglia affliction by *P. gingivalis* are listed in Table 1.

**Table 1.** Potential mechanisms for microglia affection by *Porphyromonas gingivalis (P.g.)*.

Factor	Mechanism	Ref
Gingipains	Inhibitors of gingipains are being tested for reducing p-Tau toxicity in man	[4]
	Persistent expression of gingipains in *P.g.*infected neu-rons gave AD-like neurodegeneration	[8]
	Secreted gingipains from *P.g*. induced microglia migration	[11]
	Inhibitors of Rgp and Kgp suppressed *P.g*.induced micro-glia migration	[10]
Matrix metallo-proteinases (MMPs)		
	*P.g*. can induce synthesis of MMPs in host tissues and cells	[16]
	*P.g*. may contribute to the brain pool of MMPs	[4,19]
Inhibitors of MMPs		
(TIMPs)	*P.g*. inactivated TIMP-1 and TIMP-2 causing destruction of connective tissue	[20]
*Clusterin* gene	Clusterin is a complement cascade regulatory plasma protein. *P.g*. phosphorylated its ser396 in mice	[8]
*CR1* gene	CR1 regulates complement cascade. *P.g*.causes immune subversion in relation to CR1	[52]
CD33	Belongs to an Ig-like family of receptors expressed on microglia.CR1 and is highly expressed on CD33+ cells to which *P. g*. binds	[57]
*TREM-2* gene	Codes for a protein expressed on microglia. *P.g*. down-regu-lates TREM-2 expression on microglia which may accele-rate AD	[58,59]
*TYR-OBP* gene	Key signaling molecule for *TREM-2*	[64]
Complement	*P.g*. initiated AD inflammation involving the comple-ment cascade of ApoE^−/-^ brains	[25]
LPS	Initiates neuroinflammation through microglia activation	[68]
	Migroglia were ‘primed’ inducing increased responses to subsequent challenges	[68]
	When located in brains microglia can be activated by *P.g*. LPS	[4,76]
	*P.g*. causes imbalance in the M1/M2 phenotype of microglia	[78]
Leptomeningeal cells	*P.g*. LPS stimulated transfer of inflammatory signals from peripheral macrophages to brain-resident microglia	[83,84]
	Administration of *P.g*. to mice caused *P.g*. and its prote-ases to be detected intra- and perinuclear in microglia	[8]

## Relationship between ‘chronic’ periodontitis and Alzheimer’s disease

Important in the relationship between ‘chronic’ periodontitis and AD is infection where *P. gingivalis* is a suspect pathogen, for details, see references [3–5]. An infectious episode inevitably gives rise to inflammation (albeit acute and/or longstanding) with a degree of tissue atrophy. *P. gingivalis* has several virulence factors to promote brain inflammation and associated damage.

### Gingipains

Institutionalized AD subjects show all forms of dental diseases (amongst them caries and periodontal disease), to co-exist in their dentition, and good oral health practices are unlikely to be a priority in their daily lives [6]. Recent knowledge of gingipains as virulence factors of *P. gingivalis* has initiated therapy towards reducing p-Tau peptide-related toxicity [4]. This is being tested clinically by neutralizing *P. gingivalis* virulence with inhibitors of gingipains (GAIN Trial: Phase 2/3 Study of COR388 in Subjects with Alzheimer’s Disease. ClinicalTrials.gov Identifier: NCT03823404) [4,7].

Gingipains of *P. gingivalis* are reported to digest the normal tau protein into nine fragments [4], and some of these peptides are from tau residues prone to phosphorylation and some are from two of the four microtubule-binding domains that also lie within peptides that form paired/straight helical filaments constituting NFTs [4]. This may be one pathway to releasing fragments of the tau protein into the brain’s parenchymal tissues. The extracellular phosphotau fragments generated by gingipains may be directly toxic to other neurons or the tau fragments may be of the size that neurons are able to take up at synaptic clefts during neurotransmitter uptake, thereby causing its spread from neuron to neuron. Further research is needed to clarify the role of gingipains fragmented tau peptides in the pathogenesis of AD.

#### P. gingivalis infection promotes tau phosphorylation

Gingipains have been found to be neurotoxic *in vivo* and *in vitro*, having detrimental effects on tau [4]. The capsular serotype K1 *P. gingivalis* W83 strain has shown the potential to contribute to tau phosphorylation at Ser396 in the *in vivo* wild-type mouse model [8]. Furthermore, an *in vitro* neuronal cell line model reported by Haditsch et al. [9] demonstrated an increased tau phosphorylation at Thr231 following *P. gingivalis* infection with persistent gingipain expression. Liu et al. [10] observed in their *P. gingivalis-*-infected microglial cells towards the site of infection, activation of the phosphoinositide 3-kinase/Akt (PI3K/AKT) pathway. Our own in house data show that purified *P. gingivalis* lipopolysaccharide (LPS) application to a neuroblastoma cell line, *in vitro* cell model also activated the PI3K/AKT pathway in which glycogen synthase kinases-3 beta (GSK-3β) mRNA expression increased. The importance here is that GSK-3β is one of the enzymes that phosphorylates tau suggesting that *P. gingivalis* plays an important role in the NFT lesion formation and subsequent pathophysiology of AD.

#### P. gingivalis infection promotes neurodegeneration

As mentioned, Haditsch et al. [9] reported AD-like neurodegeneration in *P. gingivalis* infected neurons in an *in-vitro* culture system with persistent expression of active gingipains. Following infection with live *P. gingivalis* (ATCC 33277) 25% of the neurons were lost in a time-dependent manner. Full-length tau was reduced in surviving cells with an increase in phosphorylation over time. This finding was related to loss of neuronal synapses and was comparable to features of associated neurodegeneration together with the presence of gingipains in AD autopsy brains. Accordingly, *P. gingivalis* can invade and survive in neurons and generate intra-neuronal gingipains that are proteolytically active, leading to neurodegeneration associated with AD.

Nonata and Nakanishi [11] found in an *in vitro* study that secreted gingipains from *P. gingivalis* induced microglial cell migration. This was likely achieved through endosomal signaling by protease-activated receptor 2 (PAR 2).

Liu et al. [10], attempting to clarify the potential effects of the gingipains – Rgp and Kgp on the cellular activation of brain-resident microglia in mice, found that Rgp and Kgp cooperated thereby contributing to the migration of *P. gingivalis*-infected microglial cells towards the site of infection, and initiated expression of proinflammatory mediators by activating PAR 2. The mitogen-activated protein kinase/extracellular signal-regulated kinase (ERK) kinase/ERK pathway contributed to both cell migration and invoked an inflammatory response in microglia. Furthermore, PI3K/AKT pathway mRNA expression increased together with pro-inflammatory mediators such as IL-6, TNF-α and inducible nitric oxide synthase. The mRNA expression of the anti-inflammatory mediators interleukin 10 (IL-10), arginase-1 and IL-4 was not affected. The authors proposed that microglial cell migration was likely to have been associated with actin polymerization and may be necessary for invoking inflammatory responses in microglia following activation of PAR 2. Further observations by Liu et al. [10] suggest and that Rgp and Kgp gingipains may be responsible for degrading components of the epithelial cell basal membrane, which may be facilitating the invasion of *P. gingivalis* into the brain. Liu et al. [10] experimentally tested their hypothesis by incorporating inhibitors of Rgp – KYT1 and Kgp – KYT36 and found the *P. gingivalis*-induced microglia cell migration was suppressed in the presence of the activated PAR 2 pathway. This provided proof of principle indicating that Rgp and Kgp were largely responsible for inducing migration of microglia in the brain. In the study by Dominy et al. [4] synthesized small-molecule inhibitors targeting gingipains were tested and this resulted in a reduction in the bacterial load. Furthermore, the small-molecule inhibitors of *P. gingivalis* reduced the extent of the brain infection established in mice. In addition, the small-molecule inhibitors blocked Aβ1-42 production, diminished neuroinflammation, and rescued neurons in the hippocampus.

### Matrix metalloproteinases

Matrix metalloproteinases (MMPs) have an important role in neuroinflammatory disorders including AD [12,13]. Increase in the expression of MMPs in the brain tissue and blood of demented patients is reported to be part of the overall inflammatory process in AD [14]. Mroczko et al. [15] detected MMP-3 and MMP-9 localized around NFTs and Aβ plaques in AD brains. Healthy elderly with increased risk of developing AD had increased levels of MMP-3 and MMP-9 protein levels in the cerebrospinal fluid. Mroczko et al. [15] proposed that increased protein levels of these MMPs may be related to neuronal degeneration and/or formation of NFTs prior to clinical cognitive deterioration [13]. Further research is required to clarify this observation.

It is accepted that *P. gingivalis* can induce the synthesis of MMPs in tissues and cells of the host. For example, cytokine and MMP expression in fibroblasts from peri-implantitis lesions were reported to be induced by *P. gingivalis* [16]. In addition, the sustained upregulation of inflammatory mediators and MMP-1 was suggested to play a role in the pathogenesis of peri-implantitis [16]. In oral squamous cell carcinoma, *P. gingivalis* promoted invasion by induction of proMMP-9 and its activation [17]. It was suggested that *P. gingivalis* activated protease-activated receptor 4 (PAR4) signaling pathways, causing proMMP-9 over-expression and invasion of oral squamous carcinoma cells [18]. Since *P. gingivalis* does spread to the AD brain as shown experimentally in mice and in humans [4,19] it is plausible to suggest that *P. gingivalis* could contribute to the pool of MMPs in the brain.

### Inhibitors of matrix metalloproteinases

Tissue inhibitors of metalloproteinases (TIMPS) can modulate the activity of MMPs [15]. This is important because dysregulation of MMPs can lead to several disorders. In a study by Sato et al. [20], sonicated *P. gingivalis* extracts caused the destruction of connective tissue, contributing to the process of periapical disease by activating pro-MMP-2 as well as by inactivating TIMP-1 and TIMP-2. In another study using human gingival fibroblasts, *P. gingivalis* LPS differentially modulated the expression of MMP-1, −2, and −3 and TIMP-1 [21]. Alternatively, there is a possibility that *P. gingivalis* suppresses TIMPs activity in the brain, to dysregulate the MMP pool in AD patients. Again further research is warranted to clarify this possibility.

## Relationship between microglia and Alzheimer’s disease

**Figure 1.**
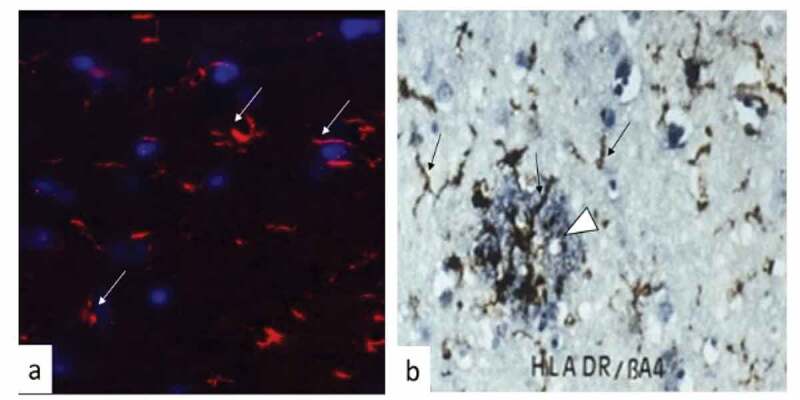
Brain tissue showing microglia responding to infection in a mouse model and to Aβ plaque in a brain tissue section from Alzheimer’s disease.

Microglia comprise 10% of the total brain cells. They are resident macrophages and the brain’s primary innate immune cells responding to diverse stimuli (Figure 1(a,b)). They also act as inflammatory cells by rapidly changing morphology, proliferating and migrating to the site of infection/injury where they phagocytize and destroy invaders and remove damaged cells. Microglia secrete cytokines, chemokines, prostaglandins, nitric oxide and reactive oxygen species [22]. During aging, they develop a more inflammatory (activated) phenotype possibly due to having previously confronted diverse antigens [23], following which they may fail to return to their original resting (non-activated) state. Some microglia can survive for long periods, even more than two decades [24]. Thus, the microglial cell population in the human adult brain is characterized by a slow turnover. Their efforts to resolve any inflammatory response involves the production of anti-inflammatory cytokines such as IL-10. In the case of experimental oral infection with *P. gingivalis* in apolipoprotein E^−/-^ (ApoE^−/-^) mice, the host releases copious amount of IL-10 in the serum. However, the bacterium itself still spreads to the brain and encounters microglia, which as a result become activated [25]. It appears that peripheral IL-10 mediated immune resolution in the ApoE^−/-^ mice remains inadequate for microglia to return to the resting state [25,26]. Recent research in mice has shown that microglia are able to ‘remember’ a previous inflammatory challenge and become ‘trained’ or ‘tolerant’ to toxins like LPS [27]. This may prolong the existence of the endotoxin in infected brains. Thus, immune training can inadvertently increase cerebral β-amyloidosis while tolerance may decrease it. The immune memory affects the reaction of microglia to new stimuli and the way in which they deal with toxic Aβ plaque in the brain (Figure 1(b)), thereby modifying neuropathology.

(a) Confocal image. Brain tissue showing microglia (white arrows) following mono-*P. gingivalis* infection (24 weeks) from an apolipoprotein E^−/-^ mouse brain immunolabelled to demonstrate microglia (anti-1ba1); Blue = DAPI; Red = TRITC label for immunopositive microglia. (b) Immunohistochemistry. Double labelling of a cortical human AD brain tissue section showing activated microglia brown (anti-HLADR) demarcated by black arrows, and Aβ plaque (anti-Aβ) blue (white arrow head) to demonstrate their relationship.

It seems the severity of AD and its progression may be linked to chronic inflammation [28,29]. It is therefore plausible to suggest that immunological memory in long-living microglia can represent a risk for not only protracting but also initiating clinical AD at the appropriate age, particularly if they become tolerant to inflammation [27]. Singhrao et al. [30] proposed that the long-term effect of inflammatory mediators, pathogens, and/or their virulence factors could, over time, prime the brain’s microglia in individuals with inherent susceptibility traits.

Microglia are not uniform cells, and this is why only fragments of microglia are seen in tissue sections following their immuno/fluorescence/histochemistry reactivities (Figure 1(a,b)). Activation of microglia in the central nervous system involves two opposing phenotypes denoted M1 and M2. Depending on the trigger that activates these two phenotypes, microglia (M1) can exert cytotoxic (proinflammatory cytokine release) or neuroprotective (M2) (immune resolution) effects [31]. Hammond et al. [32] performed single-cell RNA sequencing and *in situ* brain mapping, and detected nine transcriptionally distinct stages of >76,000 microglial cells in mice expressing unique sets of genes. Some of these genes were upregulated in microglia-surrounding Aβ plaques [33]. Microglial cell phenotypes were most diverse in the developing brain and following aging and injury. Furthermore, RNA sequencing revealed that the expression of genes from microglial cell activation increased in several neurological diseases including AD [34].

Microglia can also develop functional defects as seen in other neurodegenerative disorders [35]. During the early stages of AD, they play a key role in the clearance of Aβ and reducing the plaque burden [36]. However, Aβ plaques and extracellular tau peptides can eventually become surrounded by glial cells with dysfunctional homeostatic control and as a result acquire a proinflammatory phenotype amplifying neuronal damage [37]. Similarly, cytokines and proinflammatory molecules secreted by microglia that initially have a neuroprotective role can subsequently become the cause of further neurodegeneration [37]. If microglia become overactive, they can initiate the biosynthesis of complement proteins and with the appropriate trigger, activate the complement cascade [38]. This can lead to their aberrant digestion of nerve synapses [39]. This is observed in mice lacking the TAR DNA-binding protein 43 (TPD-43) [40]. A complement-microglial axis has been found to drive synapse loss in AD [41] and is a plausible issue for deteriorating memory.

## Relationship between *P. gingivalis* and microglia

It is noteworthy that gingipains have been detected in microglia [8], and in the capillaries of the hippocampus in ApoE^−/-^ mice brains that were from a mono-*P. gingivalis*-infected group [42]. Mice brains have shown a potent microglial activation response to mono-*P. gingivalis* infection [25]. However, there are also several other effects that *P. gingivalis* may exert on microglia, which are less appreciated.

### Affliction by genetic factors

Genome-Wide-Association Studies (GWAS) have identified several susceptibility genes expressed by microglia in AD. Genome-wide meta-analysis of clinically diagnosed AD and AD-by-proxy (71,880 cases, 383,378 controls) found associated genes to be strongly expressed in immune-related tissues (spleen and liver), and cell types such as microglia [43]. Other GWAS and integrated network studies identified immune-related pathways, as risk factors for AD, with microglia as central players. These studies strongly support the idea that genes, pathogens and the immune system act together in the eventual development of AD [5,44]. Among the genes related to microglia and AD were clusterin (apolipoprotein J), complement receptor 1 (CR1), CD33, triggering receptor expressed on myeloid cells-2 (TREM-2) and tyrosine kinase binding protein (TYR-OBP) [45]. These genes play a role in the clearance of cellular debris from the brain. However, in the context of *P. gingivalis* infection of the brain, the impressive immune subversion of this bacterium in cleaving receptors challenges this very function as discussed below.

#### Clusterin gene

The *clusterin* gene was identified as an important risk locus for AD with the three SNPs (rs 11136000, rs 2279590 and rs9331888) showing a statistically significant relationship with the disease [46,47]. Clusterin (CLU) is one of the complement cascade regulatory plasma proteins that significantly increases during AD [48]. It is present in Aβ plaques where it binds to insoluble amyloid peptides and interacts with Aβ40 and Aβ42 [49]. Due to its well-accepted role in the complement cascade, CLU is likely to affect Aβ clearance, amyloid deposition and subsequent neurotoxicity [50]. CLU is also said to stimulate expression and secretion of various chemotactic cytokines such as TNF-α, which has a critical role in promoting macrophage chemotaxis via the Pi3K/Akt mitogen-activated protein ERK and JNK pathways [51].

#### CR1 gene

It has been reported that SNPs rs3818361 and rs6656401 of the *CR1* gene are associated with increased likelihood of AD [49]. This supports a *CR1* gene defect in AD [5]. CR1 helps with regulating the complement cascade and promotes phagocytosis of cellular debris and Aβ plaques, and adherence of immune complexes to erythrocytes [5]. Interestingly, *P. gingivalis* mediates immune subversion in relation to CR1 [52]. This may suggest that a vulnerability-axis exists, within a protein region (e.g. CR1), which is exploited by both genetic defects and pathogens like *P. gingivalis*.

#### CD33

CD33 appears to have an important role in Aβ clearance and other neuroinflammatory pathways in the brain aided by microglia [50]. CD33 belongs to an immunoglobulin (Ig)-like family of receptors that are expressed on myeloid cells including microglia [53,54]. CD33 binds to alpha2-3- or alpha2-6-linked sialic acids (N-acetyl neuraminic acid) to which *P. gingivalis* also binds [55]. Sialylation of *P. gingivalis* cell surface components such as LPS may provide additional beneﬁts to this prominent periodontal pathogen in bioﬁlm formation and in escaping complement-mediated killing [56]. CR1 is highly expressed on CD33+ cells which facilitate *P. gingivalis* binding to them and is also a general clearance receptor for pathogens [57]. However, *P. gingivalis* is either able to cleave CD33 from the surface membrane of cells or down-regulate functional cell surface receptors on myeloid cells. If this was the case, then the CD33 receptor expression would be affected in a similar way on microglia.

#### TREM-2 gene

The *TREM-2* gene codes for a protein in the brain that is expressed on microglial cells and is also involved in removing degenerated tissue, including remnants from neuroinflammation [58]. The TREM-2 protein has been found to increase the susceptibility to AD, with an odds ratio similar to that of the apolipoprotein *ε4* allele [59,60]. TREM-2 deficiency inhibited Aβ degradation in a primary microglial culture and in a mouse brain model [61]. Interestingly, *P. gingivalis* significantly down-regulated TREM-2 expression in microglia [62]. Lack of TREM-2 protein may accelerate aging processes, neuronal cell loss and reduce microglial activity leading to neuroinflammation [63].

#### TYR-OBP gene

*TYR-OBP* has been identified as a key regulator among genes involved in phagocytosis [64]. It is a key signaling molecule for *TREM-2*, as determined from networks involved in immune and microglia-specific modules disrupted in AD brains [64]. The association of this gene defect with *P. gingivalis* activity is little understood. Further research is needed to clarify if *P. gingivalis* can affect *TREM-2* signaling through *TYR-OBP*.

### Complement activation

*P. gingivalis* has been proposed to exploit complement receptors 1 and 3 for evading innate immune clearance [65,66]. Active invasion of *P. gingivalis*-induced complement activation in ApoE^−/-^ mice brains has been investigated [25]. Microglia in both infected (*P. gingivalis*, oral infection) and control groups exhibited strong intracellular labeling with complement components/opsonins from C3 and C9, due to ongoing biosynthesis and activation. Further, Poole et al. [25] showed that *P. gingivalis* was able to access the ApoE^−/-^ brain and contribute to the development of AD inflammatory pathology through mechanisms involving acute-phase proteins, cytokines and the complement cascade where neurons would be attacked. It has since been shown that ApoE binds to activated C1q and that the resulting C1q-ApoE complex becomes a common player to affect brain inflammation [67]. Thus, inappropriate complement activity plays a significant role in AD pathophysiology. Interestingly, treatment with small interfering RNA (siRNA) against C5, which is formed in all complement pathways, attenuated Aβ-associated microglia accumulation [67]. As mentioned, microglia and the complement-dependent pathway can over-prune functional synapses and lead to memory loss [45].

### Activation by lipopolysaccharide

LPS is one of the major virulence factors of *P. gingivalis*. Several animal studies have shown that LPS administered directly into the peritoneum of the brain initiates neuroinflammation in the form of microglial cell activation [e.g. 68]. Researchers measured the inflammatory response following LPS administration in experimental mice and this demonstrated learning and memory impairment in test mice [69,70]. In the Cunningham study [68] the microglial cells were ‘primed’ so that they induced increased inflammatory responses to subsequent LPS challenges.

In a study by Henry et al. [71] peripheral LPS challenge in aged mice induced a hyperactive microglial response together with a higher induction of inflammatory IL-1β and anti-inflammatory IL-10. Injection of LPS caused a marked induction of mRNA expression of both IL-1β and IL-10 in the cortex of aged mice as compared to adults. An age-dependent increase in the major histocompatibility complex (MHC) class II mRNA and protein expression was also seen in microglia, suggesting their activated status. Other studies have indicated that peripheral injection of *P. gingivalis* LPS also causes a higher increase in IL-1β. Interestingly, the most prominent induction of IL-1β was detected in MHC II (+) microglia from aged mice [72]. In another study, Zhang et al. [73] found that *P. gingivalis* LPS induced cognitive dysfunction, mediated by neuronal inflammation via activation of the TLR4 signaling pathway in C57BL/6 mice. Both microglia and astrocytes in the cortex and hippocampus were activated. Accordingly, age-associated priming of microglia seems to have a central role in exaggerated inflammation induced by activation of the peripheral immune system. IL-1β is also implicated in synaptic loss [74,75], promoting deterioration in cognition [45] by stimulating Aβ cleavage indirectly from the action of cathepsin B on the APP with its cognate receptor (IL-1 R) on neurons [72]. Last, but not least, *P. gingivalis* LPS has been reported in the human brain, thus suggesting it might activate brain microglia participating in brain inflammation [4]. This idea was supported in an 18-h *in vitro* stimulation study with ultrapure *P. gingivalis* LPS in rats that resulted in classical and alternative activation of rat brain microglia and the concomitant release of cytokines and chemokines [76].

Microglia, being influenced by their environment, can assume a diversity of phenotypes and can change functions aimed to maintain homeostasis. Like their macrophage ‘cousins’, microglia show unique features with regard to phenotype polarization. As mentioned, they can be stimulated by LPS and IFN-ɣ to develop into an M1 phenotype for expression of proinflammatory cytokines, or by IL-4/IL-13 to an M2 phenotype for resolution of inflammation and tissue repair [77]. Whether *P. gingivalis-*LPS has this capacity is not known. *P. gingivalis* causes an imbalance in M1/M2 activation in macrophages, resulting in a hyperinflammatory environment that promotes the pathogenesis of periodontitis [78]. These authors reported that *P. gingivalis* or *P. gingivalis*-derived LPS-induced inflammatory responses enhanced M1 macrophages and suppressed M2 macrophages, even in the presence of IL-4. Interestingly, resveratrol has been found to reduce inflammatory damage and promote microglia polarization to the M2 phenotype in LPS-induced neuroinflammation [79].

### Transduction of inflammatory signals to microglia by leptomeningeal cells

The leptomeninges (pia mater and the arachnoid together housing the brain and spinal cord) plays a role as secretory cells, which transduce systemic inflammatory signals into the CNS [80–82]. In studies by Liu et al. [83] and Wu and Nakanishi [84] leptomeningeal cells transduced inflammatory signals from peripheral macrophages to brain-resident microglia exposed to *P. gingivalis* LPS. The mean amount of TNF-α and IL-1β after exposure to conditioned medium from *P. gingivalis* LPS-stimulated macrophages were significantly higher than after treatment with *P. gingivalis* LPS alone. This indicated that leptomeningeal cells could transduce inflammatory signals to microglia in the deeper brain areas, which in turn initiated neuroinflammation.

### Porphyromonas gingivalis DNA in brain microglia

Repeated chronic oral administration of *P. gingivalis* in wild-type mice transferred *P. gingivalis* to the brain where the bacterium and its proteases (gingipains) were detected within intra-nuclear and peri-nuclear locations of microglia, astrocytes, neurons, and extracellular spaces [8]. Microgliosis and astrogliosis were found in the experimental but not in the control group, and significantly higher levels of expression of IL6, TNF-α and IL-1β were detected in the experimental group. Also, neurodegeneration was more evident in the experimental group. Extracellular Aβ42 was detected in the parenchyma of the experimental group but not in controls. This was the first report of p-Tau (Ser396) and NFT formation. Ilievski et al. [8] have proven the concept that chronic periodontal infection can result in the formation of the diagnostic neuropathology lesions consistent with AD. Haditsch et al. [9] confirmed the findings of Ilievski et al. [8] for p-Tau on Ser396 and additionally demonstrated an increased tau phosphorylation at Thr231 following *P. gingivalis* infection with persistent gingipain expression with ongoing neurodegeneration.

## Concluding remarks

GWAS have indicated that genes, pathogens and the immune system act together to generate AD. In addition, neuroinflammation plays a pivotal role and this has made scientists ask the question if AD is an infectious disease. In this complex interaction of different players, microglia seem to be important in the host defense against invasion of the keystone periodontopathogen *P. gingivalis*. The latter may affect microglia in both direct and indirect ways. Whether other putative periopathogens and even intestinal bacteria also affect microglia of the AD brain remain to be tested. Astrocytes, which are macroglia, can also be activated by *P. gingivalis*. Such activation may have toxic effects on neurons. The chronic nature of low-level infections such as ‘chronic’ periodontitis and associated byproducts, e.g. endo/exotoxins and cytokines could affect susceptible brains’ defense capacity to a point where microglia involved in brain protection become adversely affected. Whether microglia will ‘remember’ inflammation caused by *P. gingivalis* and develop ‘tolerance’ to it, requires further research. However, it is plausible to suggest that once microglia are primed by *P. gingivalis* exposure, there remains the possibility of developing tolerance through the mastery of innate immunity manipulation by this bacterium, which may be the result of inadequate clearance of cellular debris (Aβ) from the AD brain.
